# Factors affecting recruitment into General Practice: a double binary choice approach

**DOI:** 10.1007/s10459-019-09938-w

**Published:** 2019-11-06

**Authors:** Ian Davison, Chris McManus, Celia Brown

**Affiliations:** 1 School of Education, University of Birmingham, Edgbaston, Birmingham, B15 2TT UK; 2 Psychology (Bedford Way), University College London, London, UK; 3 Warwick Medical School, University of Warwick, Coventry, UK

**Keywords:** Career choice, Cohort study, General Practice, Path analysis, Recruitment, Specialty selection

## Abstract

Recruitment to General Practice (GP) is currently low in many countries. Here we focus on two binary choices for junior doctors: first, whether to apply to GP; second, whether to accept a GP training place if offered. Previous attitudinal studies have indicated factors claimed to affect recruitment. The current study goes further by quantifying the relative impact of different factors on the propensity of candidates to apply to GP and accept a training place. An online questionnaire was sent to candidates applying to United Kingdom (UK) specialty training in 2015. Descriptive statistics and a path analysis evaluated the importance of various factors on GP applications. Our results were synthesised with an analysis of data from the online applications portal. With 3838 candidates responding to the survey, the path analysis showed that personality and previous GP experiences were strongly associated with the decision to apply. There was some evidence that it was easier to enter GP than other specialties; in terms of deciding whether to accept, the evidence suggests GP was a backup plan for around 9% of candidates who accepted a GP post. Our results indicate that recruitment initiatives should focus on candidates who apply to GP but not as first choice or consider GP but do not apply, particularly by providing substantial experience of GP and accentuating the positives of the specialty such as work-life balance and the intellectual challenge of working with patients in primary care. Acceptance of a GP place may also depend on competition for places in other specialties.

## Introduction and literature

The problem of insufficient recruitment of doctors to primary care is widely reported around the globe, including the European Union (OECD and European Union 2018), US (Kozakowski et al. 2017), Norway (Kjosavik 2018) and particularly into rural areas within Canada (Asghari et al. 2017) and Australia (McGrail et al. 2016). In the UK, the issue has become urgent (Marchand and Peckham 2017) with GP numbers falling and insufficient recruitment (Hillier et al. 2017).

However, back in 2010, General Practice (GP) was “going through a phase of relative popularity in the UK” (Watson et al. 2011 p. e645) when recently qualified GPs and trainees in the UK reported high levels of satisfaction with 95% of those saying they would choose a medical career again indicating that it would be in General Practice. The most common reasons given were ‘compatibility with family life’, followed by ‘challenging medically diverse discipline’ and ‘the one-to-one care General Practice offers’. In contrast, a more recent large-scale national survey reported that only 47% of current GPs “would recommend a career in General Practice to an undergraduate or a doctor in training” (British Medical Association 2015, p. 6). These GPs rated variety of work, relationships with patients over time, and no requirement to do out-of-hours as the most attractive aspects of their role; negative aspects related to workload and lack of time with individual patients. For GP trainees, poor work-life balance, low morale and negative portrayal of GP in the media are cited as negative influences on their career plans (Dale et al. 2017). Further upstream, although newly qualified doctors valued the work-life balance, pay and job stability provided by GP, they were deterred by uncertainties in these and the perceived stigma of being a GP (Merrett et al. 2017). More positively, in a survey across seven European countries, ‘compatibility with family life’ and ‘challenging medically broad discipline’ were the reasons most commonly cited for choosing GP by trainees and those newly qualified (Roos et al. 2015).

In terms of recruitment, a key factor is likely to be the relative attractiveness of GP compared to other career options. A review by Shadbolt and Bunker (2009) suggests that work-life balance is a positive motivator, but perceived lack of intellectual challenge is a negative.

Lambert et al. (2012) note that very few reject GP after seriously considering it, those that do mainly cite ‘job content’ such as being monotonous or isolated; therefore they conclude that the GP recruitment problem is that many doctors do not consider it as a career choice at all.

A somewhat dated literature review of 73 articles from the United States and Canada found that length of experience in family practice was most strongly associated with choosing primary care (Bland et al. 1995). More recently, Marchand and Peckham (2017) included 36 studies in their review of the literature on GP recruitment and retention. In terms of maximising recruitment, they emphasise “early exposure to primary care practice, the fit between skills and attributes, and a significant experience in a primary care setting” (ibid e227) and they go on to indicate that intrinsic factors like job satisfaction are more important than external rewards including higher pay.

The above literature gives both system level and personal explanations of why junior doctors may or may not choose to enter General Practice. Bland et al. (1995, p. 622) couched these explanatory variables in terms of students or junior doctors trying “to match the characteristics of a specialty, as he or she perceives them, with his or her career needs, including personal needs, societal needs, and the need to meet the expectations of others”. Equally important are the selection decisions made by the different medical specialties. There is some evidence that those entering GP have lower qualifications entering medical school than for other specialties (McManus et al. 2003). Therefore, some doctors may only enter GP if they are unable to secure a post elsewhere. Over the last few years, these doctors may be more successful at obtaining non-GP posts as the number of specialty posts has been rising and the number of applications falling (British Medical Association 2017).

The literature relating to medical career choice is largely based on self-report using questionnaires and interviews; there are also some longitudinal studies investigating the impact of various factors on this career choice. However, we have found no literature focussing on the actual decision-making process. Access to the UK-wide specialty selection database (Oriel) provided an opportunity to investigate this with respect to GP, and to triangulate these findings with a survey conducted in parallel.

For those unsure whether to become a GP, they face two binary choices: first, whether to apply to GP; second, whether to accept a GP training place if offered. Therefore, the present paper seeks to address the following salient questions:

1. What factors make it more likely that a candidate would apply for GP training?
2. What does the pattern of applications, offers and acceptances indicate about the relative attractiveness of GP compared with other specialties?

## Methods

Oriel is the online portal for all applications to GP and hospital specialty training in the UK. Anonymised data from Oriel were analysed from all such applications to specialties with more than 20 accepted offers in Round 1 of 2015. Several specialties with vacancies after Round 1 had a second recruitment round; we do not consider data from Round 2 here due to: relatively small numbers of candidates; added complexity of dealing with two sets of data for individual candidates; and the additional factor that their speciality choice may be influenced by rejection in Round 1. The Oriel dataset contained demographic data such as age, gender and ethnicity. For each specialty, it indicated whether each candidate had applied and was interviewed along with their shortlisting score, interview score and outcome category. The Specialties were: ACCS Emergency Medicine; Anaesthetics; Broad Based Training; Cardiothoracic Surgery; Chemical Pathology; Clinical Radiology; Community Sexual and Reproductive Health; Core Medical Training; Core Psychiatry Training; Core Surgical Training; Emergency Medicine; General Practice; Histopathology; Neurosurgery; Obstetrics and Gynaecology; Ophthalmology; Paediatrics; Public Health; and Trauma and Orthopaedic Surgery. The outcome categories were: 1. Applied but not Shortlisted; 2. Shortlisted but withdrawn; 3. Interviewed but not Appointable; 4 Appointable but no offer made; 5. Offer made but withdrew/declined/offer elsewhere; and 6. Offer accepted. Categories 1, 3 and 4 were regarded as ‘Rejected’ and codes 2 and 5 as ‘Withdrew’. However the data do not include candidates’ preferred specialty for those making more than one application.

To calculate the percentage of candidates with two offers who accepted their GP offer, the denominator was the number of candidates accepting either offer i.e. candidates declining both offers were excluded. Similarly, to calculate relative appointability (the likelihood of being offered a post) for those applying to both GP and another specialty, the denominator was the number of candidates deemed appointable in at least one of this specialty pair i.e. those rejected by both specialties were excluded. For both these calculations, specialties with fewer than ten candidates in the numerator were excluded. The Kappa statistic was used as the measure of inter-specialty agreement on who to appoint across specialties. Note that low Kappa can be due to specialities selecting for different attributes as well as poor reliability.

In addition, a questionnaire was distributed to all candidates who had applied in Round 1, 2015. This was developed from a questionnaire used in previous years in UK specialty selection to understand candidates’ views on the application process and preferences for specialities and locations. Our modifications were designed to understand more about candidates’ reasons for and against applying to GP; extensive input from people responsible for UK specialty selection helped word the questions appropriately (note that some of these were associated with the funding of this study). All candidates were emailed information about the questionnaire and asked to complete it online using Survey Monkey. Two reminder emails were sent following the initial invitation, approximately 2 weeks apart. The questionnaire was sent following applications but before offers would have been made, to help mitigate hindsight bias. This paper focuses on responses to a selection of relevant questions including demographics, those asking for their first choice specialty, reasons for this choice, and previous experience in it. They were also asked if they considered applying to each specialty and the influence of 17 factors on applying to GP. The questions used in the analyses reported here are as follows:

- Q2. In what year did you complete your initial training (e.g. MBChB, MBBS)?
- Q3. Where did you undertake your initial medical training (e.g. MBChB, MBBS)? Response options were: UK; other EU country; and non-EU country. Note that EU = European Union. For some of the analyses, other EU country and non-EU country were combined into one category.
- Q4. Please indicate your first choice specialty in Round 1 in 2015 recruitment (Response options were: ACCS Anaesthetics; ACCS Emergency Medicine; Acute Medicine; Anaesthetics; Broad Based Training; Cardiothoracic Surgery; Clinical Radiology; Community Sexual and Reproductive Health; Core Medical Training; Core Psychiatry Training; Core Surgical Training; General Practice; Histopathology; Neurosurgery; Obstetrics and Gynaecology; Ophthalmology; Oral and Maxillo Facial Surgery; Paediatrics; and Public Health).
- Q5. Please state reason(s) for your first choice of specialty. (Please select all that apply). Response options were: My seniors/consultants advised me that I would be well suited to it; I believe that working within the specialty will allow me to provide good continuity of patient care; The financial rewards associated with specialty; Good work/life balance in specialty; Intellectual challenge of specialty; Positive experience in clinical posting in specialty; Positive experience at Medical School; Information received at Medical School; Prestige associated with the specialty; My personality is well suited to the specialty; Highly competitive specialty; Less competitive specialty—I was more likely to get a specialty training post; Less competitive specialty—I would have the option to choose where I wanted to work (geography); and Other (please specify).
- Q6. Please state your previous experience in your first choice specialty. Response options were: 1 week to 2 months; 3–6 months; 7–12 months; More than 12 months; and No previous experience.
- Q8. Did you consider applying to these Specialties? The specialties were those given in Q4, above. For each of the 19 specialties, response options were: Did NOT consider; Considered, but did not apply; and I applied.
- Q11. Please state reasons(s) for your first choice LETB/Deanery. (Please select all that apply). Response options were: Good reputation; Family/friends; It’s where I currently live; Interesting/enjoyable location; I have strong personal reasons for needing to be in this region; Training reputation; and Other (please specify). Note that LETB/Deanery refers to UK regions.
- Q12. How important were specialty and location on deciding which applications to make? Response options were: Specialty only; Specialty Slightly; Equal; Location Slightly; and Location only.
- Q13. Did you receive any careers advice to help inform your decision on which specialty/specialties to apply for? (Please select all that apply). Response options were: Senior trainees and/or consultants; Educational supervisor; Postgraduate Clinical Tutor; College Tutor; Careers websites; Foundation Programme events (careers workshops); None; Other (please specify). Note that in the UK, doctors undertake the 2 year Foundation Programme on graduating from Medical School, prior to applying for specialty training, including GP.
- Q14. “As you will be aware, the NHS (National Health Service) needs 50% of trainees to become GPs. Please indicate the extent to which the following factors influenced your decision whether or not to apply to GP training”. The 17 factors were: Advice from my seniors/consultants; The patient care that I could provide; The financial rewards associated with specialty; Work/life balance; Intellectual challenge of specialty; Experience working in GP; GP experience at Medical School; Information, advice and comments received at Medical School; Prestige associated with GP; How my personality is suited to GP; Highly competitive specialty; Less competitive specialty—I was more likely to get a specialty training post; Less competitive specialty—I would have the option to choose where I wanted to work (geography); Image of General Practice portrayed in the media; Family expectations; The requirement to take computer-based tests; Other aspects of the GP selection system; and Other (please specify). For each of these factors, response options were: Influence AGAINST applying; No influence; and Influence towards applying.
- Q22. Gender with response options of Female and Male.
- Q24. Ethnic Group with response options that we collapsed into White and BME (Black and minority ethnic).

*Statistical analysis of survey data* Conventional statistical analyses were carried out in SPSS 25.0, and path modelling in LISREL 9.30. The outcome measure in the path analysis was called “Interest In GP” and defined as: 0 = Did NOT consider; 1 = Considered but did not apply; 2 = Applied, but not as first choice; and 3 = Applied as first choice. These are derived directly from Q8, with the addition that candidates who put GP as their first choice in Q4 were coded 3. Explanatory variables included the demographic variables of gender (Q22), ethnicity (Q24), year of graduation (Q2), and place of graduation (Q3, UK: not UK). To select further explanatory variables, the items in Q14 were regressed onto “Interest in GP”, except experience of GP in medical school and at work, which were of primary interest and included anyway in the model; those items which accounted for at least 1% of the variance in “Interest in GP” were retained.

Path modelling can be regarded as a subset of structural equation modelling (Raykov and Marcoulides 2006, pp. 3 and 77) with only measured variables, latent variables being absent. The path models in the present study are hierarchical; these could be fitted using conventional multiple regression (Kenny 1979, p. 58), although here LISREL was used to allow overall goodness of fit testing. Path models in LISREL were fitted by specifying variables with regression coefficients in the BETA (B) matrix (which is subdiagonal if the model is hierarchical), and variances and covariances of variables in the PSI (Ψ) matrix and all other matrices were set at default values. BETA and PSI are LISREL’s names for the upper-case Greek letters. Following Kenny (1979), the hierarchical ordering of the variables was first determined a priori and variables were placed in a causal order from left to right. A saturated model was fitted initially in which all variables are caused by all possible variables that occur at an earlier causal level. Variables at the same causal level (i.e. demographic variables and the five specific reasons for choosing GP) were modelled as blocks of covariances in the PSI matrix, and again initially were saturated within blocks. Fitting proceeded by removing all variables which did not reach statistical significance, set at *p* < 0.001 due to the large number of participants. The final model had all paths at least at that statistical level; modification indices for omitted paths confirmed that inclusion of the variables would not have achieved that level. Overall goodness of fit was described in terms of the Chi square goodness of fit statistic (although it is sensitive to sample size), as well as the Goodness of Fit Index (GFI), the Comparative Fit Index (CFI), the Tucker–Lewis Index (TLI) (called the Non-Normed Fit Index, NNFI in LISREL), and the Root Mean Square Error of Approximation (RMSEA). GFI, CFI, TLI(NNFI) and RMSEA are all popular choices for assessing fit (Raykov and Marcoulides 2006), with recommended cutoff criteria of > 0.95, > 0.95, > 0.95 and < 0.06 (West et al. 2012). All indices except the GFI impose a penalty for model complexity. Standardised (beta) regression coefficients were used as path coefficients in the path diagram.

The STROBE checklist (von Elm et al. 2007) has been used in the reporting of this survey; however, the study design was not included in the title and no sensitivity analyses were undertaken. The full survey and further methodological details are provided in the study report (Davison et al. 2016).

## Results

In Round 1, 2015, there were 16,340 applications from the 11,782 candidates on the Oriel database, which equates to a mean of 1.39 applications per candidate, with 8572 (73%) applying to just one specialty. Of these, 4837 applied to GP; these candidates made slightly more applications on average: a mean of 1.59 applications per candidate. Note that the UK’s national application system only permits one application to each specialty by a candidate; so the number of applications per candidate will be much higher in countries where this is not the case. There were 3838 survey respondents, representing a response rate of 32.6%; of these, 1748 applied to GP, which is a 36.1% response rate. These figures can be compared with a mean response rate of 38% for online surveys of health professionals reported in a meta-analysis (Cho et al. 2013), but this mean is declining over time so our response rate is about average.

Survey respondents were broadly representative of the Oriel cohort in terms of gender (60% survey respondent and 56% on Oriel were women), ethnicity (of survey respondents, 51% reported being white British and 22% Asian or Asian British, whilst on Oriel, 48% were white British and 25% Asian or Asian British) and place of graduation (80% of survey respondents indicated being UK graduates compared with 77% on Oriel) when non-responses were ignored; however, 11% of survey respondents did not disclose their gender and 15% did not disclose their ethnicity making these comparisons difficult.

### What factors make it more likely that a candidate would apply for GP training?

This section uses the survey data. Respondents were asked for reason for their first choice of specialty (Q5). Compared with those applying to all other specialties as first choice (N = 2636), those applying to GP as first choice (N = 1202) were much less likely to do so because of the “Intellectual challenge of specialty” (29% for GP and 69% other specialties), and “Positive experience in clinical posting in specialty” (32% GP and 66% others) and more likely to do so because “I believe that working within the specialty will allow me to provide good continuity of patient care” (68% GP and 33% other specialties) and most dramatically “Good work/life balance in specialty” (90% GP and 34% other).

Those applying to GP as their first choice had less experience in their chosen specialty (Q6) than those applying to other specialties (Mann–Whitney U test, *p* < 0.001). This is partly due to 53% (138/262) of candidates who graduated from countries outside the European Union and chose GP as their first choice reported no GP experience compared to just 11% (25/230) applying to other specialties; the same trend but a smaller difference is seen in the UK with 15% (124/854) of UK GP candidates and 6% (122/2194) non-GP specialty candidates with no reported experience. At the other end of the experience spectrum, only 5% (45/854) of UK first choice GP candidates have more than 6 months experience in the specialty compared with 37% (813/2194) for UK non-GP specialties; comparable figures for graduates from outside the European Union are 44/262 = 17% for GP and 120/230 = 52% for non-GP specialties.

Respondents were asked about the sources of careers advice to help inform their application decision (Q13). A lower percentage of those applying to GP as their first choice specialty indicated that they received careers advice from all six sources compared to candidates to other specialties, so that 27% of GP and 14% of non-GP first choice candidates indicated that they have received no careers advice from these sources.

When asked “How important were specialty and location on deciding which applications to make?” (Q12), those with GP as their first choice gave slightly more weight to location than those with other first choice specialties (Mann–Whitney U test, *p* < 0.001). Compared with non-GP first choice candidates, a slightly higher percentage of their reasons for location choice (Q11) was “Family/friends” (26% vs. 22%) and “It’s where I currently live” (25% vs. 20%), and a lower percentage for “Good reputation” (12% vs. 16%) and “Training reputation” (8% vs. 13%).

### Path modelling of reasons for applying for General Practice

Initial regression retained five items from Q14, each of which accounted for at least 1% of the variance in Interest in GP; these were: “The patient care that I could provide”; “Work/life balance”; “Intellectual challenge of specialty”; “How my personality is suited to GP”; and, “Image of General Practice portrayed in the media”, which we will refer to as “Reasons”.

**Fig. 1:**
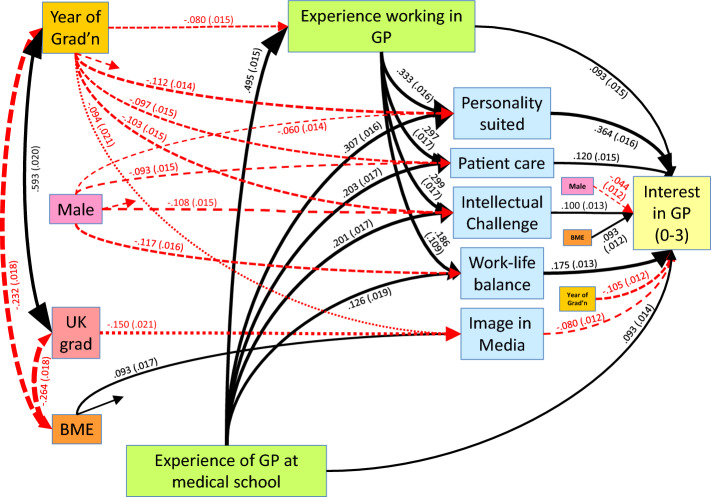
Fitted path model of predictors of applying for General Practice

With “Interest in GP” as the outcome measure, the path model included the five Reasons, the two measures of experiences of General Practice (“GP experience at Medical School” and “Experience working in GP”), and four demographic variables (Year of Graduation, Male, UK graduated and BME)—see Fig. 1. Causal ordering followed the approach of Davis (1985). Temporal ordering means that the four demographic variables are extrinsic to the system, and therefore placed to the far left. Likewise, “Interest in GP” is the outcome measure and is at the far right. The five Reasons are immediately proximal to Interest in GP, being reasons for the interest, and therefore are immediately to the left of Interest in GP. “GP experience at Medical School” must occur before “Experience working in GP” since one necessarily occurred before the other, and both occurred before Reasons were elicited for being interested in GP. That therefore gives an a priori causal ordering which makes educational sense. As with any causal ordering, other possibilities can be considered, but those in Fig. 1 provide a principled way of thinking of the relations between the twelve variables and constraining their causal ordering.

Figure 1 shows the path model fitted using LISREL. Overall the model fits the data well, with CFI = 0.997, TFI (NNFI) = 0.993, and GFI = 0.998 all meeting the recommended values of > 0.95 (West et al. 2012). RMSEA = 0.0176 (90% confidence intervals: 0.0106–0.0244) is substantially lower than the recommended level of < 0.05, also indicating a good fit (Raykov and Marcoulides 2006). The maximum likelihood Chi square statistic (C1) = 51.474 (df = 25) is significant (*p* = 0.0014), but is difficult to make non-significant with large sample sizes (Raykov and Marcoulides 2006): with N = 3428, the fit can be regarded as good.

Figure 1 displays the fitted path model. Arrows signal the assumed direction of causation (but see discussion, below), wider arrows designating stronger effects, and dashed, red arrows representing negative effects. Standardised path coefficients (betas) along with standard errors are shown alongside arrows. On the left are the four demographic variables; the thin arrows indicate relatively weak relationships with the other variables. However, the patterns are generally consistent with more recent graduates, males and white candidates all tending to have less positive interest in becoming a GP, both directly and indirectly (note that only the start and finish of the arrows representing these direct effects are displayed). Having positive experiences of GP at both medical school and in work have only very small direct associations on “Interest in GP”, but there are stronger indirect associations mediated via the top four Reasons for becoming a GP. Of these variables, “How my personality is suited to GP” is by far the strongest factor, followed by “Work/life balance”, then “The patient care that I could Provide” and “Intellectual challenge of Specialty”; “Image of General Practice portrayed in the media” has only a small negative association. The negative coefficient for the latter item is because the media image of GP was an influence against applying for only 37% of those who did not consider applying compared with 44% for all other applicants.

**Fig. 2:**
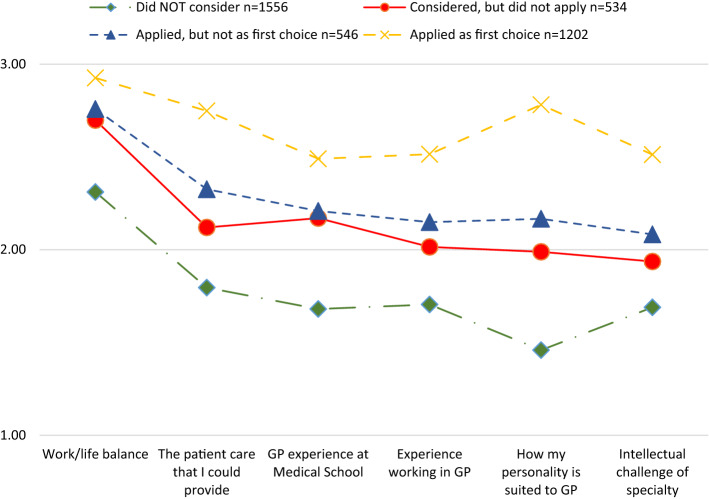
Mean ratings for 6 question items for the 4 levels of *Interest in GP*

Figure 2 presents the mean ratings of the 6 questions most strongly associated with Interest in GP; on the y-axis, 1 = Influence AGAINST applying; 2 = No influence; and 3 = Influence towards applying. Questions on the x-axis are ordered by overall mean score, with “Work/life balance” being the most positive and “Intellectual challenge of specialty” least positive. Kruskal–Wallis one-way ANOVAs undertaken separately on the 6 questions were all statistically significant, *p* < 0.001; post hoc Mann–Whitney U tests between “Considered, but did not apply” and “Applied, but not as first choice” are non-significant for “Work/life balance” and “GP experience at Medical School”; the significance level of “Experience working in GP” was *p* = 0.005; “Intellectual challenge of specialty”, “How my personality is suited to GP” and “The patient care that I could provide” were all significant at *p* = 0.001 or better. Despite these significant differences, visually it is notable that mean ratings on all 6 questions were pretty similar for these two groups (differences in means range from 0.04 to 0.21). Generally there are much larger differences between “Applied as first choice” and “Applied, but not as first choice” (range 0.17–0.61) and between “Considered, but did not apply” and “Did NOT consider” (range 0.25–0.53).

### What does the pattern of applications, offers and acceptances indicate about the relative attractiveness of GP compared with other specialties?

This section uses the Oriel data. One frequently-used measure of difficulty of obtaining a post is the competition ratio (the number of applications per post available), which for the UK are at https://specialtytraining.hee.nhs.uk/Competition-Ratios. Table 1 shows the number of applications per accepted offer, which is a slightly different form of competition ratio because not all posts may be filled. As candidates who withdraw are not providing any competition for the posts available, the final column has excluded these withdrawals for a stricter measure of competition. Table 1 is ordered by number of offers accepted. General Practice is the largest speciality in terms of offers accepted and has the lowest ratio of non-withdrawn applications per accepted offer at 1.49; however the next six largest specialities are not that dissimilar as they all have ratios of less than 2. This competition ratio is greater than 2 for all of the seven smallest specialties in Table 1.

**Table 1:** Specialty competition ratios

		Rejected by specialty					Rejected by candidate		
Specialty	Total applications	Applied but not Shortlisted	Interviewed but not Appointable	Appointable but no offer made	Offer accepted	Applications per accepted offer	Shortlisted but withdrawn	Offer made but withdrew/declined	Non-withdrawn applications per accepted offer
General Practice	4837	407	591	210	2477	1.95	638	514	1.49
Core medical training	2531	125	350	251	1328	1.91	105	372	1.55
Anaesthetics	1273	42	211	287	623	2.04	22	88	1.87
Core surgical training	1433	49	464	77	600	2.39	49	194	1.98
Paediatrics	814	47	89	174	430	1.89	10	64	1.72
Acute care common stem—emergency medicine	884	75	165	97	359	2.46	23	165	1.94
Core psychiatry training	690	56	89	132	317	2.18	13	83	1.87
Clinical radiology	936	88	237	189	239	3.92	134	49	3.15
Obstetrics and gynaecology	632	45	114	178	238	2.66	26	31	2.42
Ophthalmology	372	18	137	23	94	3.96	85	15	2.89
Public health medicine	712	115	96	51	87	8.18	351	12	4.01
Histopathology	203	25	80	5	71	2.86	5	17	2.55
Broad based training^a^	344	46	46	59	59	5.83	72	62	3.56
Neurosurgery	175	16	9	34	30	5.83	72	14	2.97

^a^Consisting of core medical training, GP, paediatrics and psychiatry

Table 2 displays information on the applications of candidates who applied to GP and another specialty sorted by the percentage of the other specialty’s candidates who also applied to GP. As GP is the largest specialty, multiple applications are always a greater percentage of the other specialty’s applications than of GP; for example, 42% of Core Psychiatry candidates applied for GP, whereas just 6% of GP candidates applied for Core Psychiatry.

**Table 2:** Multiple applications including General Practice

Specialty	Total applications to other specialty	Applications to both GP and other specialty	% GP applications^a^	% other specialty applications
Broad based training	344	241	5.0	70.1
Core psychiatry training	690	292	6.0	42.3
Histopathology	203	68	1.4	33.5
Clinical radiology	936	277	5.7	29.6
Ophthalmology	372	109	2.3	29.3
Obstetrics and gynaecology	632	165	3.4	26.1
Core medical training	2531	644	13.3	25.4
Acute care common stem—emergency medicine	884	212	4.4	24.0
Paediatrics	814	189	3.9	23.2
Public health medicine	712	127	2.6	17.8
Core surgical training	1433	202	4.2	14.1
Anaesthetics	1273	168	3.5	13.2
Neurosurgery	175	18	0.4	10.3

^a^4837 applied to GP

**Fig. 3:**
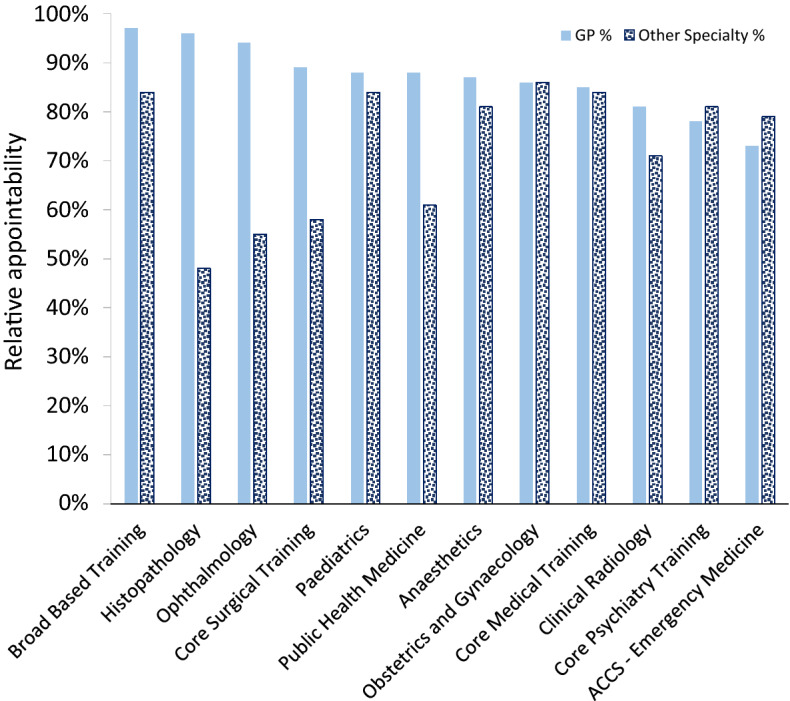
Relative appointability of candidates applying to GP and another specialty

These multiple applications allow us to consider whether it is easier to obtain a training place in GP than in other specialities. Figure 3 displays the percentage of offers received from GP and other specialties when candidates have applied to both and received at least one offer. These are ordered by the percentage receiving GP offers. The biggest difference was with Histopathology; of those receiving at least one offer, 96% received a GP offer and 48% a Histopathology offer. In two specialties, Acute Care Common Stem (ACCS)—Emergency Medicine and Core Psychiatry, candidates were less likely to receive an offer for GP. Overall, 85% of these candidates were offered posts in GP and 79% in the other specialty. The overall Kappa score was 0.40 (45% of maximum Kappa given numbers appointable).

To consider the relative attractiveness of specialties, data were analysed from those candidates who were made offers by both GP and another specialty and then accepted one of these offers. In total there were 454 such candidates across nine non-GP specialties, of whom 173 (38%) chose General Practice and 281 (62%) chose the non-GP specialty. The percentage accepting their GP offer ranged from 12.5% with Clinical Radiology to 57% with Obstetrics and Gynaecology, which was the only specialty where the percentage accepting their GP offer was greater than 50%.

Of the GP accepted offers, 591/2477 = 24% were for candidates who had applied to one or more other specialties. To avoid complex cross-tabulations, the analysis was restricted to the candidates (N = 444/591, 75%) who only applied to one other specialty. Table 3 displays the number of candidates who were rejected, withdrew, and accepted a post in GP and their other specialty, for those candidates who applied to exactly two specialities including GP. Of those who accepted one post and withdrew from the other, 42% (163/163 + 222) displayed a preference for GP, and 58% (222/163 + 222) preferred the other specialty. (Similarly, 44% (192/436) of survey respondents who applied to GP and one other specialty had GP as their first choice.) The only candidates who may have accepted GP not as their first choice are the 281 who accepted a GP post whilst being rejected by the other specialty. If we assume these candidates had the same ratio of preferences as those who withdrew from one specialty and accepted the other, then about 58% of these would have had the other specialty as their first choice. Therefore an estimate of the number accepting a GP offer not as first choice is 58% of 281 = 162 candidates who applied to GP and one other specialty.

**Table 3:** Outcomes for candidates applying to GP and one other specialty

*n *= 1216		Other specialty applications		
		Rejected	Withdrew^a^	Accepted post
General Practice applications	Rejected	162 (13%)	43 (4%)	150 (12%)
	Withdrew^a^	122 (10%)	73 (6%)	222 (18%)
	Accepted post	281 (23%)	163 (13%)	0

^a^Note that some withdrew before interviews, so it is unknown whether they would have been offered a post

This equates to 216 (162*591/444) candidates if this logic holds for those accepting a GP post after applying to 3 or more specialties, which is 8.7% (216/2477) of accepted GP offers.

## Discussion

This paper considers the factors that affect the double binary choice facing candidates who may be recruited into General Practice; first, whether to apply; second, whether to accept an offer if given. We have used survey data to consider reasons for applying to GP and application data from Oriel to investigate candidates’ acceptances of offers, particularly those who applied to GP and another specialty.

Regarding the first choice, those applying to GP in this cohort were more likely than other candidates to cite continuity of patient care, intellectual challenge and particularly having a personality suited to GP as important in their career choice. GP candidates also reported receiving less careers advice. Despite recent concerns about deteriorating work-life balance in GP, this was a major reason for choosing it compared with other specialties, which aligns with increased importance associated with location and choosing the specialty for personal rather than reputational reasons.

The path analysis evaluated the importance of these factors on candidates’ propensity to apply for GP training, suggesting that the biggest factors influencing doctors’ interest in GP were positive experience of GP at medical school and afterwards as well as perceiving that GP suited their personality, provided work/life balance, was good for providing patient care and intellectual challenge. These six factors all discriminate between those who applied to GP as first choice and those who did not consider applying (Fig. 2): this suggests that there are substantial differences in experience and attitudes between these two groups so that it would be difficult to change their propensity to apply to General Practice. However, there was relatively little experience and attitudinal difference between those who applied to GP not as first choice and those who considered GP but didn’t apply; therefore it may be possible to encourage this latter group to apply to GP as a backup plan. These findings contrast with the claim that “the biggest recruitment losses are those not ever considering General Practice as a career” (Shadbolt and Bunker 2009, p. 55). Although in absolute numbers our findings concur regarding this first binary choice, we contend that the candidates who apply to GP not as first choice or consider GP but do not apply should be the focus of any recruitment initiatives. As positive experiences of GP at medical school and afterwards are such big determinants of GP applications, it is noteworthy that GP candidates report far less experience in the specialty than other candidates. One obvious reason for this is that almost all GP placements in the UK Foundation Programme are in Year 2, so many will have already applied for specialty training before their GP experience. Similarly, medical schools with more General Practice teaching have a greater percentage going onto GP (Alberti et al. 2017).

We turn now to the second binary choice of whether to accept a GP offer, which concerns the relative attractiveness of GP. Analysis of the Oriel dataset indicates that GP did have a low competition ratio in 2015, but that this is expected given that it is by far the largest specialty and this ratio is not that different from the other large specialties. General Practice makes slightly more offers than other specialties overall for those candidates who apply to GP and another specialty (85% vs. 79% relative appointability). This could be taken as evidence that ‘the bar’ is slightly lower in GP; alternatively, junior doctors with multiple applications including GP may be more suited to GP than their other specialty of application. Elsewhere we have combined the Oriel data described above with Foundation Programme application scores: on average, GP candidates’ scores are lower than for all other large specialties apart from ACCS Emergency Medicine (Davison et al. 2016). The Kappa score for those with multiple applications, described above, is at the low end of ‘moderate’ agreement (Viera and Garrett 2005), implying that either the specialties have substantially different requirements or there is unreliability in the selection processes. Candidates applying to GP make more applications to other specialties than non-GP candidates; when candidates were given two offers including GP, only about 40% chose General Practice; these findings lend credence to the possibility that the majority of these candidates are using GP as a backup plan. Overall, the slightly low competition ratio, slightly greater offer ratio, lower Foundation Programme scores and lower acceptance rate from those with multiple offers all imply that GP is of below average attractiveness for those applying to specialty training.

Using the data in Table 3, it was estimated that around 9% of accepted GP offers were from candidates applying to GP not as first choice. This may be an over-estimate as candidates overall probably do better in their preferred specialties; however, if ‘the bar’ is lower in GP, then this may be an under-estimate. The data show that 76% of candidates accepting a GP post in Round 1 2015 only applied to that specialty and that a significant number accepting a GP post withdrew from another specialty; therefore the vast majority of those accepting GP posts have GP as their first choice specialty.

The survey data suggest it would be easiest for recruitment initiatives to move candidates from “Considered but did not apply”, to “Applied, but not as first choice”. However, analysis of application outcomes indicates that only a small percentage of GP posts is filled by candidates for whom GP is not their first choice. This implies that recruitment should focus on making GP the first choice for more candidates, for example by providing positive experiences in General Practice. Further research would be required into the meaning of attitudinal factors such as personality suited to GP and how they can be influenced.

These career choice dynamics will depend on numerous factors such as the relative difficulty of being appointed to different specialities. Nevertheless, it could be argued that many of these findings are likely to apply to other countries with a shortage of applications to GP; however research in these countries would be required to determine the generalizability of these findings.

Although this is a large UK-wide study using two sources of data, the 33% survey response rate, reliance on self-report for the survey and its cross-sectional design are limitations. We argue that the causal ordering in the path analysis, above, makes educational sense, for example, that positive experience of GP increases the likelihood of choosing GP as a career; however, as a cross-sectional study, we cannot discount the possibility that choice of GP as a career may influence recollection of previous GP experience. Cognitive interviews and piloting could have been used to investigate whether respondents interpret the questions as intended (Artino et al. 2018). For example, in the UK context, “Experience working in GP” is commonly understood to refer to postgraduate (and hence paid) training so can be distinguished from “GP experience at Medical School”; however, some respondents may have interpreted this distinction differently. Perhaps of most theoretical interest are the four attitudinal variables in Fig. 2. For example, regarding “How my personality is suited to GP”, a longitudinal study would be required to see if personality on entering medical school influences positive experience of GP, or if positive experience of GP means candidates feel GP is suited to their personality. Our study did not investigate what applicants meant by their personality being suited to GP, but it could be that they value work-life balance more than specialty reputation and find continuity of care more rewarding than the high adrenaline excitement found elsewhere. This view has some concordance with previous research that suggests that those choosing GP might have greater empathy with patients and be less sensation-seeking than those applying to some other specialties (Borges and Savickas 2002; Mehmood et al. 2013).

Unlike almost all career-choice literature, this study has combined self-report survey data with outcomes data; ideally the two datasets would have been linked, but anonymity requirements precluded this.

Pfarrwaller et al. (2017) conceptualise career choice within Bronfenbrenner’s ecological model such that a candidate will have been influenced throughout their career by those around them, the places where they have worked and studied, and the wider culture. These influences, their pre-existing characteristics and expectations of possible careers feed into the candidates’ career choice decisions. In their language, our current study is concerned with the decision process, based to a small extent on their pre-existing characteristics, but far more on how they perceive their interests match the characteristics of GP.

## Conclusion

Our findings support those from previous studies. Additionally, the path analysis enables estimation of the relative importance of different influencing factors, which shows the huge influence of personality and previous positive experiences of GP (Fig. 1). Those who considered but didn’t apply to GP have similar attitudes and GP experience to those who applied, but not as first choice. Therefore recruitment initiatives could focus on encouraging applications from those who are considering GP but likely not to apply. However, as candidates with GP as their first choice fill most GP posts, it can also be argued that recruitment should encourage those intending to apply to GP as a back up to view it as their first choice.

For those unsure whether to become a GP, they face two binary choices: first, whether to apply to GP; second, whether to accept a GP training place if offered. To be more likely to apply to GP: the lure of the specialty needs to be maximized by previous positive experience; they should prefer the challenges provided by primary care patients; and value the work-life balance of being a GP. To accept a GP place also depends on the availability of posts in other specialties where GP is not the candidate’s first choice.

## References

[CR1] Alberti H, Randles HL, Harding A, McKinley RK (2017). Exposure of undergraduates to authentic GP teaching and subsequent entry to GP training: A quantitative study of UK medical schools. British Journal of General Practice.

[CR2] Artino AR, Durning SJ, Sklar DP (2018). Guidelines for reporting survey-based research submitted to academic medicine. Academic Medicine.

[CR3] Asghari S, Aubrey-Bassler K, Godwin M, Rourke J, Mathews M (2017). Factors influencing choice to practise in rural and remote communities throughout a physician’s career cycle. Canadian Journal of Rural Medicine.

[CR4] Bland C, Meurer L, Maldona G (1995). Determinants of primary care specialty choice: A non-statistical meta-analysis of the literature. Academic Medicine.

[CR5] Borges NJ, Savickas ML (2002). Personality and medical specialty choice: A literature review and integration. Journal of Career Assessment.

[CR6] British Medical Association (2015). National survey of GPs: The future of general practice 2015.

[CR7] British Medical Association. (2017). ‘The state of pre and post-graduate medical recruitment in England, September 2017’. London https://www.bma.org.uk/-/media/files/pdfs/collective%20voice/policy%20research/education%20and%20training/state-of-medical-recruitment-sept-2017.pdf. Retrieved February 23, 2018.

[CR8] Cho YI, Johnson TP, Vangeest JB (2013). Enhancing surveys of health care professionals: A meta-analysis of techniques to improve response. Evaluation and the Health Professions.

[CR9] Dale J, Russell R, Scott E, Owen K (2017). Factors influencing career intentions on completion of general practice vocational training in England: A cross-sectional study. British Medical Journal Open.

[CR10] Davis JA (1985). The logic of causal order.

[CR11] Davison, I., McManus, C., & Taylor, C. (2016). ‘Evaluation of GP specialty selection’. Birmingham: University of Birmingham. https://www.ucl.ac.uk/medical-education/publications/reprints2016/GPspecSelReport.pdf. Retrieved February 23, 2018.

[CR12] Hillier M, Bacon R, Boswell P, Elphicke C, Evans C (2017). ‘Access to general practice: Progress review’ house of commons committee of public accounts.

[CR13] Kenny DA (1979). Correlation and causality.

[CR14] Kjosavik SR (2018). Ongoing recruitment crisis in Norwegian general practice. Scandinavian Journal of Primary Health Care.

[CR15] Kozakowski S, Travis A, Marcinek J, Bentley A, Fetter G (2017). Entry of medical school graduates into family medicine residencies: 2016–2017. Family Medicine.

[CR16] Lambert T, Goldacre R, Smith F, Goldacre M (2012). Reasons why doctors choose or reject careers in general practice: National surveys. British Journal of General Practice.

[CR17] Marchand C, Peckham S (2017). Addressing the crisis of GP recruitment and retention: A systematic review. British Journal of General Practice.

[CR18] McGrail MR, Russell DJ, Campbell DG (2016). Vocational training of general practitioners in rural locations is critical for the Australian rural medical workforce. Medical Journal of Australia.

[CR19] McManus IC, Smithers E, Partridge P, Keeling A, Fleming PR (2003). A levels and intelligence as predictors of medical careers in UK doctors: 20 year prospective study. British Medical Journal.

[CR20] Mehmood SI, Khan MA, Walsh KM, Borleffs JC (2013). Personality types and specialist choices in medical students. Medical Teacher.

[CR21] Merrett A, Jones D, Sein K, Green T, Macleod U (2017). Attitudes of newly qualified doctors towards a career in general practice: A qualitative focus group study. British Journal of General Practice.

[CR22] OECD & European Union (2018). Health at a glance: Europe.

[CR23] Pfarrwaller E, Audetat MC, Sommer J, Maisonneuve H, Bischoff T (2017). An expanded conceptual framework of medical students’ primary care career choice. Academic Medicine.

[CR24] Raykov T, Marcoulides GA (2006). A first course in structural equation modeling.

[CR25] Roos M, Watson J, Wensing M, Peters-Klimm F (2015). Motivation for career choice and job satisfaction of GP trainees and newly qualified GPs across Europe: A seven countries cross-sectional survey. Education for Primary Care.

[CR26] Shadbolt N, Bunker J (2009). Choosing general practice: A review of career choice determinants. Australian Family Physician.

[CR27] Viera AJ, Garrett JM (2005). Understanding interobserver agreement: The Kappa statistic. Family Medicine.

[CR28] von Elm E, Altman D, Egger M, Pocock S, Gøtzsche P (2007). The strengthening the reporting of observational studies in epidemiology (STROBE) statement: Guidelines for reporting observational studies. PLoS Medicine.

[CR29] Watson J, Humphrey A, Peters-Klimm F, Hamilton W (2011). Motivation and satisfaction in GP training: A UK cross-sectional survey. British Journal of General Practice.

[CR30] West SG, Taylor AB, Wu W, Hoyle R (2012). Model fit and model selection in structural equation modelling. Handbook of structural equation modeling.

